# Drought-induced Suppression of Female Fecundity in a Capital Breeder

**DOI:** 10.1038/s41598-019-51810-9

**Published:** 2019-10-29

**Authors:** Charles F. Smith, Gordon W. Schuett, Randall S. Reiserer, Catherine E. Dana, Michael L. Collyer, Mark A. Davis

**Affiliations:** 1 Department of Biology, Wofford College, Spartanburg, South Carolina 29303 USA; 2 The Copperhead Institute, Spartanburg, South Carolina 29323 USA; 3 Chiricahua Desert Museum, Rodeo, New Mexico 88056 USA; 4 Department of Biology and Neuroscience Institute, Georgia State University, Atlanta, Georgia 30303 USA; 5 Illinois Natural History Survey, Prairie Research Institute, University of Illinois Urbana-Champaign, Champaign, Illinois 61820 USA; 6 Department of Entomology, University of Illinois Urbana-Champaign, Urbana, Illinois 61801 USA; 7 Department of Biology, Chatham University, Pittsburgh, Pennsylvania 15232 USA

**Keywords:** Climate-change ecology, Conservation biology, Population dynamics

## Abstract

Human-induced global climate change is exerting increasingly strong selective pressures on a myriad of fitness traits that affect organisms. These traits, in turn, are influenced by a variety of environmental parameters such as temperature and precipitation, particularly in ectothermic taxa such as amphibians and reptiles. Over the past several decades, severe and prolonged episodes of drought are becoming commonplace throughout North America. Documentation of responses to this environmental crisis, however, is often incomplete, particularly in cryptic species. Here, we investigated reproduction in a population of pitviper snakes (copperhead, *Agkistrodon contortrix*), a live-bearing capital breeder. This population experienced a severe drought from 2012 through 2016. We tested whether declines in number of progeny were linked to this drought. Decline in total number offspring was significant, but offspring length and mass were unaffected. Reproductive output was positively impacted by precipitation and negatively impacted by high temperatures. We hypothesized that severe declines of prey species (e.g., cicada, amphibians, and small mammals) reduced energy acquisition during drought, negatively impacting reproductive output of the snakes. Support for this view was found using the periodical cicada (*Magicicada* spp.) as a proxy for prey availability. Various climate simulations, including our own qualitative analysis, predict that drought events will continue unabated throughout the geographic distribution of copperheads which suggests that long-term monitoring of populations are needed to better understand geographic variation in drought resilience and cascading impacts of drought phenomena on ecosystem function.

## Introduction

The onset of the Anthropocene has ushered in an epoch defined by rapid, human-induced global climate change^1^, for which profound negative effects on the health, survival, and diversity of Earth’s biota are projected^2–7^. Anthropogenic climate change is predicted to increase the incidence and severity of extreme climatic events^8–10^ and acutely alter the risk of hydrological extremes at regional scales^11–13^. Drought specifically is expected to increase in frequency, severity, and duration, with severe consequences to ecosystems^14^. Predicted impacts on biodiversity are stark, with mass extinctions expected^15–17^ and already occurring^18–21^.

The serious impacts of climate change, such as rising temperatures, drought, elevated sea-levels, pollution, and disease-risks, on amphibian populations, for example, are documented on a global scale^22–26^, and they are among the most endangered groups of vertebrates^24,25,27,28^. Following a similar fate, many populations of reptiles appear to be in decline as a result of global climate change, in particular owing to rising temperatures and drought^26,29,30^. Importantly, as ectotherms, life-history traits (e.g., maturity, fecundity, and offspring size) of amphibians^22,31–33^ and reptiles^34–36^ are strongly influenced by climate and weather, particularly temperature and precipitation^19,37^. During unfavorable conditions these taxa are especially prone to alter or entirely shut down reproduction as a trade-off^38,39^. Furthermore, for capital breeders, these responses can be exacerbated via dependency of reproductive success on energy intake acquired at earlier periods. In females of some species, this could require multiple seasons of optimal conditions to acquire sufficient energy (lipids) reserves^35,40,41^. Despite our knowledge of ectotherm life-histories, our overall understanding of responses to climate change in these taxa is poor. Why? Because it is often difficult in short-term studies to document reproductive trends and population declines in small, cryptic species^42^. Consequently, more often than not, we fail to capture onsets and transitions and only see endpoints, well after preemptive adaptive management interventions can be implemented. Unabated declines in reproductive effort over a sufficient temporal span can have serious and deleterious demographic effects—leading to extirpation of populations^22^ and eroding ecosystem services provided by biodiversity^43–48^ — yet are troublesome to document, especially in cryptic species such as snakes.

Here, in a multi-year study, we monitored reproductive output (measured by total offspring birthed) in a Connecticut population of the copperhead (*Agkistrodon contortrix*), a long-lived, live-bearing North American pitviper (Serpentes, Viperidae) that is a capital breeder^35,49,50^. The population we studied, occurring at the northeastern extent of the species’ distribution^50^, experienced drought from 2012 through 2016^51^. We tested three main hypotheses: five consecutive years of drought negatively impacted 1) total number of offspring produced in a specified year, 2) litter size (fecundity) in a specified year, and 3) offspring size (snout-vent length, body mass). Drought as we define it is inadequate precipitation in terrestrial ecosystems, generally occurring over an extended period, which depletes soil moisture and impacts all organisms^52^. Serendipitously, we initiated aspects of this research in 2011 prior to the onset of the protracted drought event in 2012 and followed through its final year (2016) and into 2018. Drought has been linked to negative effects on reproduction (e.g., litter size, size of propagules) in a variety of ectothermic vertebrates, notably amphibians^22,25,52^ and non-avian reptiles^53^, including several lineages of snakes^30,54–58^. Because many ectothermic taxa are capital breeders and thus reproduction in females is dependent on energy acquired in previous seasons^41,42,59–61^, we investigated whether a food staple of copperheads^62^, the northern 17-year periodical cicada (*Magicicada*), was linked with specific reproductive parameters in copperheads [Fig. S1]. To the best of our knowledge, similar assessments have not been performed on North American pitvipers.

## Materials and Methods

### Subjects

The copperhead (*Agkistrodon contortrix*) is a medium-sized (mean max snout-vent length ~ 1 m), terrestrial, North American pitviper^49,63^ exhibiting sexual size dimorphism (SSD), with males larger than females^49^. It is viviparous, long-lived, iteroparous, and a capital breeder^59–61,63^ that generally reproduces on a biennial cycle based on prey availability^63^. Litter size varies (5–15 offspring) but is largely dependent on female size^49,62,63^.

### The study site

**Figure 1:**
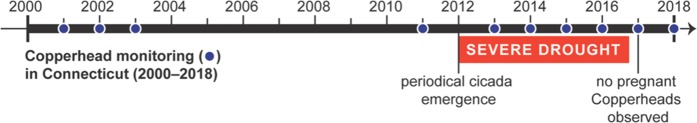
Timeline of sampling and prolonged drought at Meriden, Connecticut. Preliminary sampling occurred from 2001–2003. Full sampling began in 2011 and continued through 2018. Logistical difficulties prevented sampling in 2012. Onset of drought and a periodical cicada emergence occurred in 2012. The drought officially ended in late 2016, after the copperhead active season. The year 2017 represents the first post-drought year, and 2018 the first year following a year of normal precipitation.

Lying at the northeast edge of both the copperhead and 17-year periodical cicada (Brood II) distribution, the research site was a 485 ha parcel of basalt trap rock ridge ecosystem situated 4.75 km NW of Meriden, Connecticut [Fig. S2]. Topography is consistent with trap rock systems found throughout the Central Connecticut River Valley^61^. North and south oriented ridges, ≥200 m in elevation, are bordered to the west by steep cliff faces and extensive talus slides, and to the east by gently sloping woodlands. Two prominent basalt ridges are located at this site. Sources of water are an ephemeral pool and a 1 ha ephemeral wetland that supplies the 7.3 ha Crescent Lake, a reservoir located at the site that supplies the town of Meriden Connecticut with water; there is 1 ha drainage at the southern end of Crescent Lake. The drought period identified by the National Integrated Drought Information System (NIDIS) was from 2012 through 2016^50^. During this period the overall landscape became drier. Each spring during the drought, the ephemeral pool would fill minimally (if at all) and be dried by April. The ephemeral wetland, which typically remains wet throughout spring and summer, was consistently dry by late spring and early summer during the drought [Fig. 1]. Crescent Lake remained intact but did not support large populations of amphibians owing to the presence of the common snapping turtle (*Chelydra serpentina*) and introduced predatory game fishes (C.F. Smith, pers. observ.)

At the study site [Fig. S2] copperheads emerge from winter hibernacula (“dens”) in late March and early April, and they remain at or near these sites until early May^48,50^. Pre-ovulatory females (destined to be pregnant that year), however, remain at or near the hibernacula for the duration of summer (through gestation to birthing period). The five-month active season largely corresponds to the period from April 15 to September 15. Based on extensive field observations of this population^61,63^ there is a single mating season in late summer, females store sperm over the winter, with ovulation and fertilization occurring the following May or early June^61,63^. The birthing season is from late July to mid-September, and litters are produced in rookeries near winter shelter sites [Fig. S2] associated with their mothers^61,63^. On average, females reproduce on a biennial schedule^61,63^.

### Collection of reproductive data

Preliminary sampling was conducted from 2001 to 2003, initially to identify the location of birthing rookeries, and intensive sampling was conducted every year from 2011 through 2018. Logistical difficulties prevented sampling in 2012. Pregnant females were located in the field by searching known rookeries^61^. Over seven years (six consecutive 2011; 2013–2018) an average of 200.6 (±23.27) person-hours per year were spent each summer searching for females (4 people searching, 8 hr days for 7 days). Regression analysis revealed no significant difference in search effort among years (R^2^ = 0.26, P_1,8_ > 0.1). Over the course of the study, a total of six observers searched for snakes. Three observers (including CFS) participated in every survey, while two other observers alternated among years. For each survey period, four highly trained observers (including and led by CFS) would visit rookeries in the morning (before rookeries became too hot) and conduct both visual encounter surveys (VES) and, if necessary, inspection by fiber optic cameras to identify the presence of females. Any snakes encountered would be captured. Later in the day, VES would be conducted elsewhere on the site. Observers would also conduct VES at rookeries in late afternoon. This sequence was alternated every day for a period of 7 days. All surveys were conducted in late July through early August but varied slightly based on weather conditions.

GPS coordinates were obtained for all capture sites. From 2011 to 2016,  adult females suspected of being pregnant (*N* = 40) were collected and brought to the laboratory, processed, and provided private enclosures. Details of animal care are provided elsewhere^61,63^. In 2018, females were not collected and brought to the laboratory; rather, portable ultra-sonography was performed in the field on pregnant females (*N* = 16).

All adult female copperheads captured at the study site (SVL range = 48.0–67.8 cm) were processed for body mass (BM: ±0.5 g using an analytical balance), snout-vent length, and tail length (SVL: ±0.2 cm; TL: ±0.2 cm), using photographs imported into ImageJ image processing and analysis software^64^. Individuals were permanently marked for identification using intramuscularly injected passive integrated transponder (PIT) tags (125 kHz 12 mm, Biomark, Boise, Idaho, USA). Pregnant females were assigned a PIT-tag but remained unmarked until after giving birth.

### Laboratory observations

Pregnant females were checked for parturition multiple times daily. Within several hours following parturition, mothers and neonates were processed (mother BM: +/−0.5 g pre- and post-parturition using an analytical balance, neonates BM: ±0.0001 g using an analytical balance). Each neonate was photographed (dorsal and ventral aspects) by gentle restraint in a 9.2 cm diameter clear plastic petri-dish containing a soft, disposable sponge. Ventral snout-vent length (SVL: +/−1 mm) and ventral tail length (TL: +/−1 mm) were measured using photographs imported into ImageJ^64^. After parturition all snakes (mothers, offspring) were returned to their original capture sites using GPS data. Data on parturition and neonate size were not obtained in 2018.

### Climatic data

Climatic records (2001 to 2018) were obtained from the Meriden Markham Municipal Airport at Meriden, Connecticut (41° 30′31.3730″N; 072°49′46.1220″W), which is 7.6 km from the study site (https://www.wunderground.com/history/airport/KMMK/). Total precipitation (rainfall and snowfall) – considered both for previous year and matched year - and temperature data were used in the present analyses. Data on annual total precipitation over a 30-year period were obtained for inspection of trends [Fig. S2].

### Cicada emergence records

We identified northern 17-year periodical cicada (*Magicicada*) emergence years in CT (and other states) during the study period (http://magicicada.org/magicicada/broods/), as periodical cicada and copperhead distributions largely overlap. These emergences introduce substantial energy into the ecosystem^65^ and impact abundance in a variety of vertebrate species^66,67^. Cicadas are a major prey source for copperheads [Fig. S1] throughout their wide distribution^67,68^.

### Analysis

We used an information theoretic approach^69^ to assess the influence of environmental variables on population reproductive output of female copperheads. For each year in which sampling occurred, we determined the total annual number of pregnant females, the total annual number of offspring produced by the pregnant females, and the average annual litter size (fecundity) for pregnant females. We accessed the following variables from Meriden Markham Municipal Airport at Meriden, Connecticut: annual precipitation, annual average temperature, number of days above 32 degrees C, and number of days below 0 degrees C. We also quantified annual search effort. An initial assessment of the association between the number of pregnant females and total offspring revealed a strong linear correlation (Person’s *r* = 0.99, *t*_8*df*_ = 20.78, *P* < 0.001), meaning the mean litter size was rather consistent across years (mean ± SEM: 6.77 ± 0.24; i.e., 6–7 offspring per female). Therefore, analyzing total offspring was sufficient for considering the influence of environmental variables on recruitment.

Because copperheads are long-lived, iteroparous, capital breeders^61,64^, we used variables from the previous year for periodical cicada emergence and climate parameters (i.e. annual average temperature, annual average precipitation, number of days above 32 degrees C, and number of days below 0 degrees C) for predicting total offspring. We developed linear models for the number of pregnant females (GF), total offspring (TO), and average number of offspring per litter (AO), as dependent variables. Various combinations of independent climate variables were used as predictors, plus two other variables. One was a binary factor, drought, which simply described years as drought years or not. The other was search effort (hours). We considered three initial models for predictors: search effort only, drought plus search effort only, and all variables excluding drought. The latter two models considered whether simply classifying years as drought years or whether other variables associated with drought are better predictors; the former considered whether search effort only was the best indicator of total offspring observed. Akaike’s information criterion (AIC) was used to compare these models. We subsequently used a stepwise procedure with both forward and backward selection and AIC as a criterion to ascertain which variables among those in the best of the three models were important predictors of total offspring. Dependent variables were log-transformed, e.g., ln (TO + 1), to address the assumption of normally distributed linear model residuals. Because one year yielded no pregnant females (2017), data were removed for analysis of AO, as AO was valueless in this case.

Once we arrived at a parsimonious model, we used randomization of residuals in a permutation procedure (RRPP) with 10,000 random permutations to estimate effect sizes of model terms as standard deviates (*z*-scores) from their sampling distributions, using marginal sums of squares estimation^70^. Fitted (predicted) values were compared to observed total offspring, by year, to qualitatively evaluate model effectiveness, in addition to comparing effect sizes. All analyses were performed in R, version 3.6.0^71^ using the lm, AIC, and step functions of the stats package^58^ and the lm.rrpp function of the RRPP package, version 0.4.2^72^.

### Bioclimatic analysis

Bioclimatic variables were obtained to qualitatively inspect our drought results in context. Specifically, we generated maps that include the current North American range of copperheads, as well as three key climate variables^73^: annual precipitation, precipitation in driest month, and precipitation seasonality. These data layers were acquired for contemporary conditions and for a 2080 projection. We used the IPCC A1B emission scenario, which predicts a technological change in the energy system that yields a balance across energy sources, defined as reliance on a variety of energy sources and with the assumption that similar improvement rates apply to all energy supply and end-use technologies^71^. This scenario assumes rapid economic growth, a mid-21^st^ century population peak, and rapid introduction of new and more efficient technologies. This model provides a “best-case” climate scenario.

## Results

**Figure 2:**
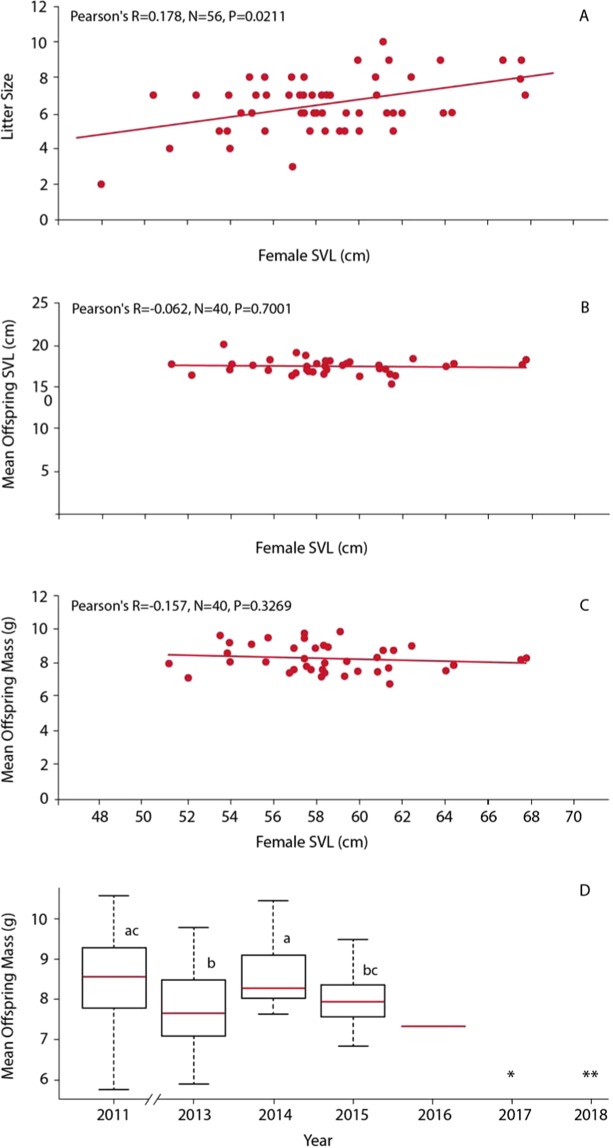
Reproductive data for female copperheads and offspring. (**A**) Female SVL (cm) and litter size for litters from 2011 to 2018. (**B**) Female SVL (cm) and mean offspring SVL (cm) for litters from 2011 to 2016. (**C**) Female SVL (cm) and mean offspring mass (g) for litters from 2011 to 2016. (**D**) Mean offspring mass (g) from pre-drought (2011) to drought (2012–2016) sampling periods (ANOVA, *P* < 0.05). Note: no pregnant females were encountered in 2017 (*), and litter size in 2018 was estimated using portable ultrasonography in the field. Hence, offspring length and mass data were not collected for 2018 (**).

Over the course of the study, there was considerable variation in the number of pregnant females, with observed peaks occurring in the year prior to the onset of the drought (2011) and again two years after the drought broke (2018). We also note that in the year following the final year of the drought (2017) no pregnant females were detected at the site (Table 1). In 2018, the first year following a return to normal precipitation, a substantial increase in the number of pregnant females (N = 16) conferred an increase in total progeny N = 100, mean = 6.25) compared with 2016 (N = 1 reproductive female, 5 total progeny) and 2017 (N = 0 females and 0 total progeny). Results of a regression analysis reveal no significant difference in mean litter size among years (R^2^ = 0.20, P_1,7_ = 0.23). Our analyses further revealed that 1) litter size increased as female size (SVL) increased, but 2) size (SVL) and mass of copperhead progeny was invariant despite female body size, levels of fecundity (litter size), and year sampled [Fig. 2]. However, standardized litter size showed some difference in drought and non-drought years (Fig. S4).

**Table 1:** Raw data for sampling years.

Year	Pregnant Females	Total Offspring	Mean Offspring	Cicada Emergence	Search Effort (hrs)
2001	3	14	4.67	0	224
2002	9	67	7.44	0	198
2003	6	33	5.5	0	210
2011	20	148	7.4	0	224
2012	N/S	N/S	N/S	1	N/S
2013	9	58	6.44	0	200
2014	3	15	5	0	220
2015	7	38	5.43	0	192
2016	1	5	5	0	196
2017	0	0	0	0	198
2018	16	100	6.25	0	144

In each year of sampling, total number of pregnant females, total offspring, and mean litter size were documented. Additionally, whether or not a northern 17-year periodical cicada emergence was coded as a binary variable. Finally, search effort (in hours) was quantified.

No variables explained variation in ln(AO + 1); i.e., a model containing only an intercept (overall mean) was sufficient, suggesting that litter size was rather consistent throughout the study period. Furthermore, because AO = TO/GF, results using either TO or GF were interchangeable (confirmed with Pearson *r* = 0.99). We therefore only present results for TO, recognizing that the number of offspring is directly proportional to the number of pregnant females.

Initial comparison of models ln(TO + 1) revealed that a model including bioclimatic variables (AIC = 32.95) performed much better than one with drought as a predictor, absent of specific variables (AIC = 39.47). A model with research effort alone was comparatively inferior (AIC = 41.09), suggesting that the total number of offspring was dependent, at least in part, on bioclimatic variables.

**Figure 3:**
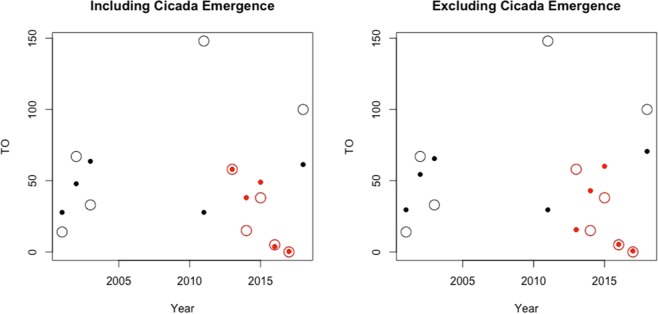
Observed total offspring by year (open circles) and fitted values from the bioclimatic model (solid dots). Drought years are represented in red; all other values in black. Fitted values are shown for models including and excluding periodical cicada emergence as a variable.

The stepwise procedure revealed precipitation in the previous year (not in the matched year), periodical cicada emergence, and number of days above 32 degrees C were the only meaningful bioclimatic variables associated with the natural log of total offspring (AIC = 31.18). Precipitation and periodical cicada emergence positively influenced and the number of days above 32 degrees C negatively influenced the number of total offspring. Although periodical cicada emergence had the largest coefficient for (positive) log of total offspring, it was not significant and had the smallest effect size (Table 2). This result is likely due to a single emergence event (2012) in our data, resulting in higher standard error for this estimate. Both precipitation and number of days above 32 degrees C has coefficients that were smaller in magnitude but were significant and had similar effect sizes (*Z*-scores, Table 1). The marginal sum of squares (*SS*) as a fraction of the total *SS* (*R*^2^) were 0.58 and 0.37 for precipitation and number of days above 32 degrees C, respectively. Removing periodical cicada emergence from the model had little influence on fitted values, other than to suggest an expected lower number of offspring in 2013, suggesting the emergence might have mitigated effects of the drought [Fig. 3]. Overall, the model fitted values (predictions) tracked observed total offspring fairly well during drought years [Fig. 3] but underestimated total offspring in the non-drought years before and after, especially 2011 when total offspring was exceedingly larger in our sample. This result pattern suggests that other factors (perhaps other prey abundance), which could also be drought-dependent, might be needed to explain increases in total offspring in non-drought years.

**Table 2:** Stepwise logistic regression model parameter estimates for log(total offspring + 1) in a year, plus ANOVA statistics. Effect sizes (*Z*-scores) and *P*-values are based on 10,000 random permutations for RRPP. Type III sums of squares (*SS*) were estimated.

Effect	*DF*	Estimate	*SE*	*SS*	*MS*	*R* ^2^	*F*	*Z*	*P*
Precipitation	1	0.059	0.016	11.569	11.569	0.578	14.253	1.604	0.008
Cicada	1	1.463	0.966	1.860	1.860	0.093	2.292	0.835	0.199
Days > 32 C	1	−0.087	0.029	7.349	7.349	0.367	9.054	1.413	0.026
Residuals	6			4.870	0.812	0.243			
Total	9			20.021

**Figure 4:**
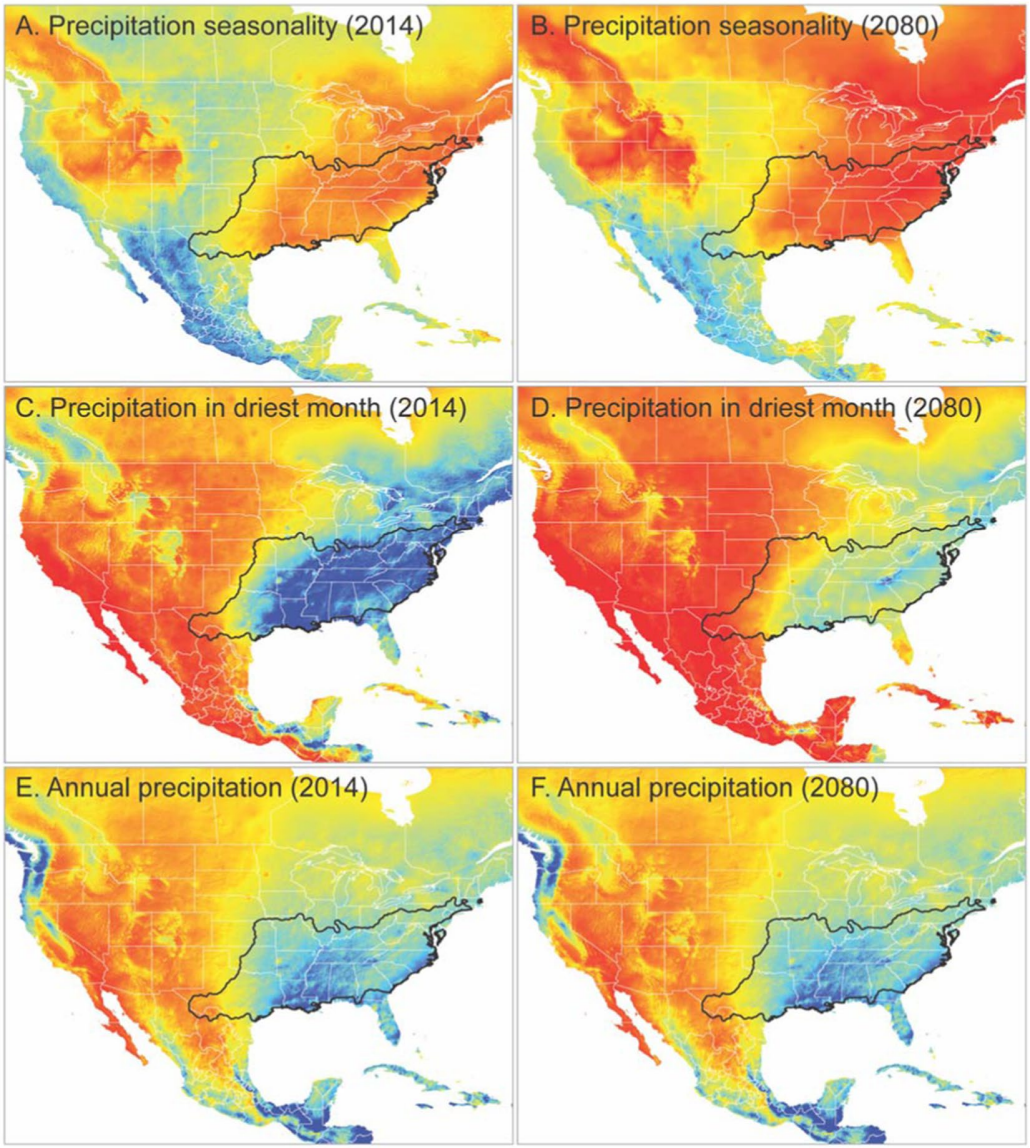
Qualitative assessment of predicted precipitation changes from 2014 to 2080 projected onto North America. Black lines on each figure delineate the range of the copperhead complex. The colder colors (e.g. blue) indicate wetter conditions, while the hotter colors (e.g. red) indicate drier conditions. Panels A–B depict the changes in precipitation seasonality (the difference between the annual maximum and minimum precipitation) for 2014 (**A**) to 2080 (**B**). Panels C–D depict the difference in precipitation in the driest month from 2014 (**C**) to 2080 (**D**). Panels E–F depict annual precipitation from 2014 (**E**) to 2080 (**F**).

Qualitative assessment of climate change projections between 2014 and 2080 reveals substantial changes in precipitation throughout the range of copperhead [Fig. 4]. A marked shift towards increased precipitation seasonality is revealed, which suggests increased precipitation variability over a four-year rolling average. In addition, we see substantial decreases in both annual precipitation and precipitation in the driest month. These changes are noted across the entire range of copperheads, but with most drastic changes at the core of the distribution.

## Discussion

Negative impacts of excessive high temperatures and depressed precipitation, hallmarks of protracted drought, on reproduction are documented in a variety of plants and animal species^8,74,75^. The region of Connecticut where we monitored copperheads in this study was declared to be officially in drought from 2012 through late 2016 and declared over in 2017^50^. Female Copperheads are capital breeders^61,64^ and at our research site generally produce litters on a biennial cycle^35,41,42,76^. Thus, in order to become pregnant, females must secure prey and gain sufficient body mass (lipid stores) in the prior year to complete vitellogenesis and produce a litter^77^. This pattern of reproduction is documented in copperheads from other geographic locations^62^ and extensively in other species of temperate pitvipers^78–82^.

Our analysis of copperheads showed that in the year following the last drought year (i.e. 2017), reproduction appears to have ceased, with depressed precipitation and abnormally high temperatures exerting significant and substantial negative impacts. This reproductive response was not unexpected based on prior research of other snake taxa^78,79^. Reducing litter size or abandoning reproduction altogether under non-optimal environmental conditions are common physiological responses or trade-offs in many plants and animal species^8,74,75^, especially in capital breeders that are long-lived and iteroparous^8,74,75^.

Outside of drought years, however, our model provided a relatively poor fit to our empirical data and underestimated reproductive output. Numerous factors could be contributing to this unexpected outcome. For example, determining frequency of reproduction is not possible for all females. It may be that under non-drought conditions, reproductive cycles are more synchronous. Furthermore, we were unable to fully reconstruct an individual female’s reproductive history, both in terms of the year of their first litter, total number of litters, and the year of their last litter. Reproductive history has been shown to be related to future reproductive output in other species^83^. Prey availability in non-drought years might have higher energetic quality, which could serve to boost reproductive output. Alternatively, complex interactions of cooler temperatures, increased precipitation, and/or other covariates may yield concomitant (positive) changes in water and prey abundance that, while not captured by our model, would increase reproductive output above the model predictions outside of drought years. Last, in periods where resources (i.e., water and prey) are particularly abundant, it cannot be discounted that additional maternal investment (in terms of resource allocation) may be supplementing capital investments^84^.

Despite the negative effects of drought on fecundity, our analysis showed that progeny size (SVL) and mass was invariant of female body size, levels of fecundity (litter size), and year sampled [Fig. 2]. Previous work on snakes has shown that survival of newborn snakes is correlated with large body size^35,83,85,86^. Accordingly, our results lend general support to the Smith-Fretwell life-history model which proposes that decreased resources for reproduction is expected to decrease litter size rather than offspring size^87–90^. Offspring size, which is critical to survival, greatly influences population demographics in long-lived species^8,91^. Based on published data on offspring size from other populations, we speculate that copperheads near the northeastern range limit (e.g., Connecticut) produce minimum-sized propagules, and a reduction in offspring size would translate to reduced maternal fitness by reducing offspring survival^89,92–94^. Therefore, in adult female copperheads, coping with an environmental stressor such as drought entails loss of fecundity but not reduction of offspring size.

Excessive high temperatures and declines in precipitation leading to drought were responsible for key changes to the landscape occupied by the copperheads, with the area overall becoming much drier after 2011. Crucially, ephemeral ponds desiccated in early spring or sometimes failed to form entirely. Lack of precipitation severely impacted aquatic plants and animals, particularly amphibians^33,53,95^. Other sources of water at the study site also diminished. Consequently, owing to water shortages and a drying landscape, we suspect there was a reduction in the recruitment and population density of amphibian species from 2012–2016. Amphibians are a major component of the copperhead diet^49,62^. Although we did not quantify the abundance of mammals, most species were observed less frequently during the drought. Rodent populations in general appeared to be noticeably less abundant following the field season in 2011. Small species, such as voles, which are consumed by copperheads^49,62^ can be negatively impacted (e.g., population declines) by drought^96^. Importantly, other predators (e.g., skunks, foxes, birds), including snake species (e.g., *Nerodia sipedon*, *Pantherophis alleghaniensis, Thamnophis sirtalis*), compete for the same prey as copperheads. Multi-trophic analyses of drought effects on reproduction in snakes are available for several species^56–59^; also for lizards^97,98^ birds^77,99,100^, and mammals^101^.

While periodical cicada emergence was recovered in the top model, effect size was small and non-significant. This disparity is likely due to the low detection of the emergence in the model. The emergence of periodical cicada occurred only once over the course of the study; however, we did not anticipate it to be significant in our linear model given the high standard error around the estimate. Nevertheless, its recovery in the top model suggests that periodical cicadas may positively impact reproductive output in this population. Periodical cicadas are clearly an important prey item for copperheads throughout their range^49,62^, [H. W. Greene, pers. comm.], and their emergences may represent an energetic boon to copperhead populations, manifest in increased reproductive output (measured in total number of offspring produced) the following year. Despite only feeding on xylem for their entire nymphal lifespan, emerged periodical cicadas have the highest recorded biomass per area for a terrestrial animal^102^. Consequently, they play an important role in large scale nutrient cycling in ecosystems^103,104^ and are a substantial food source for insectivorous birds^105^, mammals^106,107^, and fish^106^, in addition to copperheads and other reptiles. Unlike most annual cicadas, periodical cicadas emerge *en masse* and serve as a resource pulse in their emergence year^108^. Alarmingly, broods are increasingly being extirpated, particularly at the northeastern extent of the range which may compound the negative impacts of climate change on copperhead populations. Several potential causes have been proposed for reductions in *Magicicada* brood ranges and population sizes, including anthropogenic effects like changes in land use and climate change^109,110^.

We recognize that lack of water, however, may be of equal, if not greater importance as prey in capital reproduction. Pregnancy in pitvipers increases evaporative water loss^111^, placing an additional constraint on reproductive females and potentially influencing reproductive trade-offs (see below). Moreover, a study on Northern Pacific Rattlesnakes (*Crotalus oreganus*) revealed female snakes that received supplemental hydration exhibited better body condition and gave birth during the study, while snakes that received no water supplements were in poorer condition and did not give birth^112^. Finally, in the Western Diamond-backed Rattlesnake (*C. atrox*), dehydrated snakes receiving a meal in lieu of water reached severe dehydration far sooner than snakes that did not receive a meal^113^. These findings indicate that free-standing water is essential for water balance and homeostasis, certainly under extremely xeric conditions. We note that our study site, even in drought, is considerably more mesic, may experience more rain events, and may provide better hydric refugia for snakes. Thus, direct effects of water scarcity may be lessened in this system. Nevertheless, water constraints appear to be of critically important consequence to capital breeding in pitvipers^114,115^.

Despite no changes in our search protocol (i.e., number of person-hours searching each year), we documented a sharp decline in the number of pregnant females located (i.e., from 20 in 2011 to 1 in 2016, to zero in 2017; see Fig. S4), with concomitant reductions in total offspring produced, effectively eliminating annual recruitment. Whether this reflected cryptic behavior (e.g. use of subterranean shelters to reduce water loss)^116^ or mortality^59^ could not be substantiated; however, search effort remained consistent over the duration of the drought and thereafter. The drought was officially over in 2016^50^, 2017 marked a return to “normal” precipitation, and our field season in 2018 revealed a substantial increase in the number of pregnant females and total progeny (N = 16 females and 100 total progeny, mean = 6.25 per female) when compared to 2016 (N = 1 female and 5 total progeny) and 2017 (N = 0 females and 0 total progeny). Moreover, three of the 16 females in 2018 were recaptures, providing *prima facie* evidence that direct mortality of females may not have been responsible for the absence of pregnant females. While mortality remains a possible outcome^117,118^, it appears that recruitment rapidly recovered concomitant with the end of the drought, supporting the view that organisms showing capital breeding modes exhibit trade-offs between reproductive effort and future reproductive output^119,120^, but see also^81^.

Our qualitative assessment of climate reveals changes in precipitation that align with more intensive climatic modeling analyses^121^ in which drought events, particularly in the eastern United States^121^, are predicted to occur more quickly and with greater severity^122–124^. These shifts will likely exert negative impacts on a range of ecosystem functions. Particularly concerning are effects on forested systems, which copperheads occupy^50,63^, with potential increases of insect pests, pathogens, and invasive plant species, plus alteration of microclimates and precipitation cycles^125–127^. Our life history analysis also depicts indirect impacts of drought on copperheads, and we suspect that increasing severity and duration of drought could negatively impact the persistence of the present population and others at the northern extent of the distribution. Crucially, long-term ecological planning will be required to better understand the types of drought mitigation protocols needed to prevent catastrophic demographic changes leading to extirpation and loss of biodiversity^97,128,129^.

Climatic conditions, water availability, and extreme temperatures in particular place considerable selective pressures on terrestrial vertebrates, ultimately driving functional adaptations necessary to persist in variable and changing systems^115^. The synergistic impacts of multiple stressors in the Anthropocene suggest that extinction risks are greater than previously estimated^130^. Indeed, short-range endemic species have been shown to be particularly vulnerable^40,131,132^. Increasingly, however, evidence indicates that species with large distributions may be vulnerable, particularly at the edges of their ranges^133^. Extinctions at the northeast extent of the periodical cicada range have already been documented^109,134^.

Species are predicted to respond in three ways to climate change: spatially, temporally, and physiologically^4,135–139^. Spatial^5,132^ and temporal changes^140–142^ have received extensive attention. Research on the impacts of climate change on physiology, however, has been scant and more difficult to parse, despite an urgent need^143^, but see also^144^. Here we report the impacts of prolonged drought on reproductive output in a capital breeding pitviper, and mark the physiological trade-offs required to weather prolonged drought. In conclusion, our analysis provides *prima facie* evidence that common species of little current conservation concern, such as the copperhead, have an important role in understanding the population dynamics of abundance that cannot necessarily be understood in rare species^145^. In many cases, common species tend to be the most important drivers of ecosystem functions^145,146^. Although they are considered a common reptile and listed as least concern by the IUCN^147^, our analysis indicates that copperheads may be increasingly at risk of local extirpation at the edge of their range due to a myriad of stressors with manifold effects^16^.

### Ethics

Protocols for all animals used in this study were approved by local permits and the supervision of The University of Connecticut Institutional Animal Care and Use Committee (IACUC), protocol number S211-1201, and Wofford College Institutional Animal Care and Use Committee (IACUC), protocol number 802. All methods were performed in accordance with the relevant guidelines and regulations.

## Supplementary information

Supplementary Files

## Data Availability

Data supporting our results are provided in electronic supplementary material.
