# Supplementary material for: Drought-induced Suppression of Female Fecundity in a Capital Breeder
Source: Sci Rep. 2019 Oct 29;9:15499. doi: 10.1038/s41598-019-51810-9 (PMC6820553; doi:10.1038/s41598-019-51810-9)
Supplement: Supplementary file 1 — Supplementary Files [file 41598_2019_51810_MOESM1_ESM.pdf]

Drought-induced Suppression of Female Fecundity in a Capital Breeder

Charles F. Smith, Gordon W. Schuett, Randall S. Reiserer,
Catherine E. Dana, Michael L. Collyer and Mark A. Davis

Electronic Supplementary Information

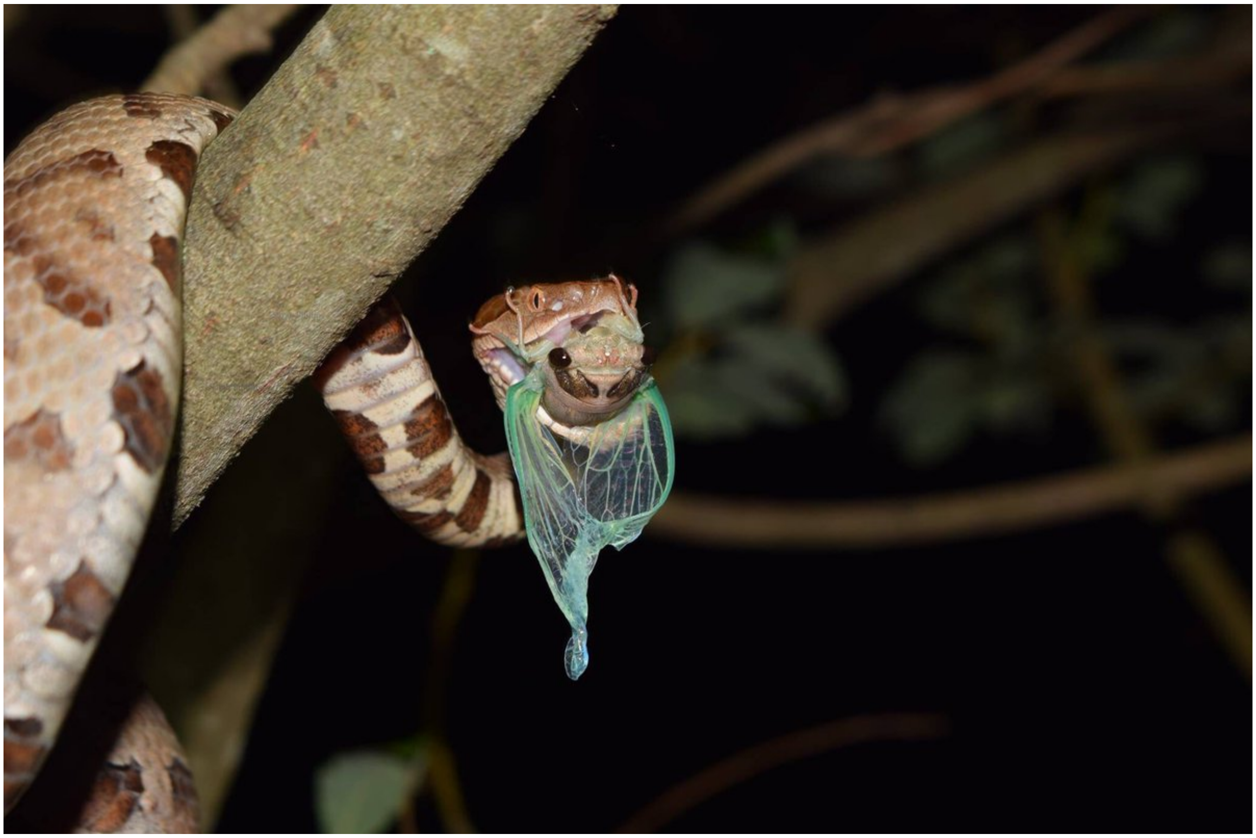

Figure S1. An adult copperhead (*Agkistrodon contortrix*) consuming an emerging cicada. Photo: Sarah Phillips.

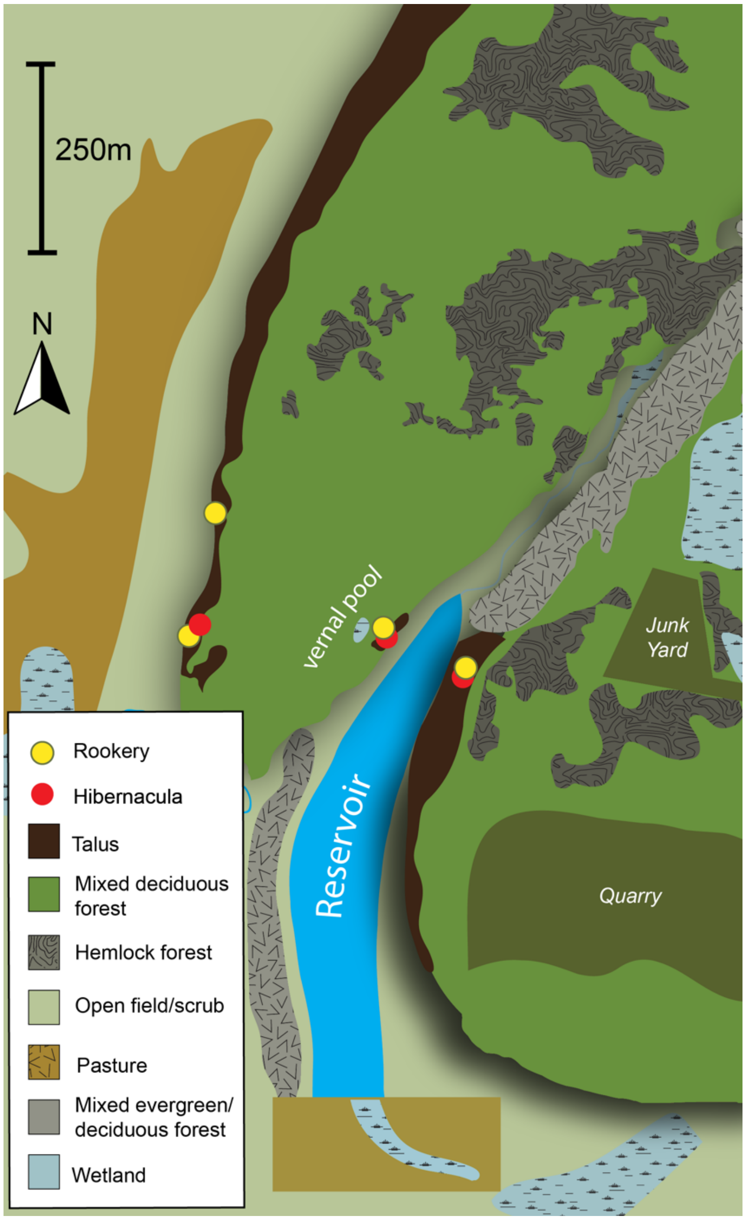

Figure S2. Schematic of research site at 4.75 km NW of Meriden, Connecticut (New Haven Co.)

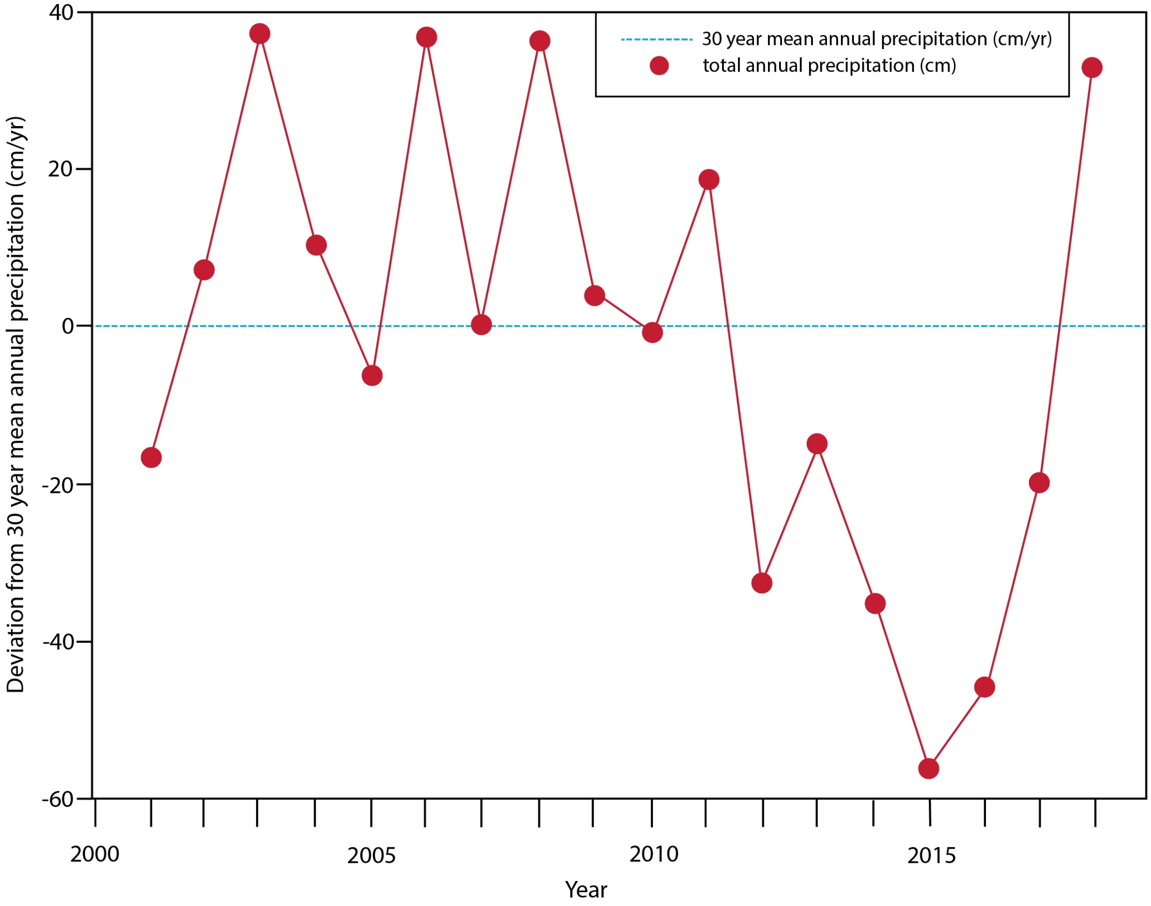

Figure S3. Deviation of the total annual precipitation observed for the years 2001 to 2018 (red circles) from the 30-year mean total annual precipitation (1981-2010, dashed line, mean = 100.7 cm/yr) recorded at the Markham Municipal Airport, Meriden, Connecticut [(KMMK) 41°30’31.3730”N; 072°49’46.1220”W; 7.6 km from the study site)]. Deviation from the 30-year mean was calculated by subtracting the observed total annual precipitation for each year (2001 to 2018) from the 30-year mean; thus, any year with a total annual rainfall close to the 30-year mean would approach zero. For 11 years prior to and including 2011, nine of those years were near or above the 30-year mean total annual precipitation. From 2012 to 2017, precipitation values were below the 30-year mean total annual precipitation.

Mean annual precipitation data for Meriden, Connecticut for the 30-year period from 1981 to 2010 were obtained online through the NOAA National Centers for Environmental Information (https://www.ncdc.noaa.gov/data-access/land-based-station-data/land-based-datasets/climate-normals/1981-2010-normals-data). Total annual precipitation data or the years 2001 to 2018 were obtained online through the weather history search function on Weather Underground (https://www.wunderground.com/history/airport/KMMK/) and recorded at Markham Municipal Airport, Meriden, Connecticut.

Standardized Litter Size and Drought

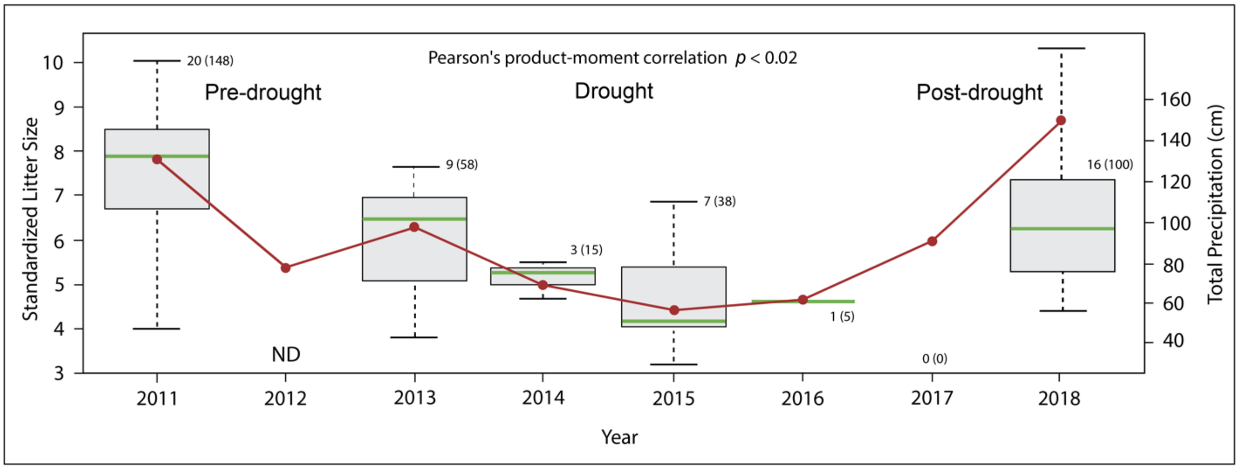

Figure S4. Box-and-whisker plots. Standardized litter size in female copperheads (n = 56 litters, 364 offspring). Litter size was significantly correlated with total annual precipitation from 2011 to 2018. No pregnant females were located in 2017. Median (green line, Q_2), upper quartile (75th percentile, Q_3), lower quartile (25th percentile, Q_1), upper extreme, lower extreme. Values beside the upper extreme denote total number of litters in a given year; parenthetic values denote the total number of progeny. ND = no litter data. Red circles denote total annual precipitation. Total annual precipitation for this site over a 30-year period (1981-2010) is provided [electronic supplementary material, figure S3]. Analysis follows: Peig, J. and Green, A. J. New perspectives for estimating body condition from mass/length data: the scaled mass index as an alternative method. *Oikos*, *118*(12), pp.1883-1891 (2009).
